# Supplementary material for: COVID-19-Related Vaccine Hesitancy among Community Hospitals’ Healthcare Workers in Singapore
Source: Vaccines (Basel). 2022 Mar 30;10(4):537. doi: 10.3390/vaccines10040537 (PMC9032808; doi:10.3390/vaccines10040537)
Supplement: Supplementary file 1 [file vaccines-10-00537-s001.zip › vaccines-1645832-supplementary.pdf]

Supplementary file

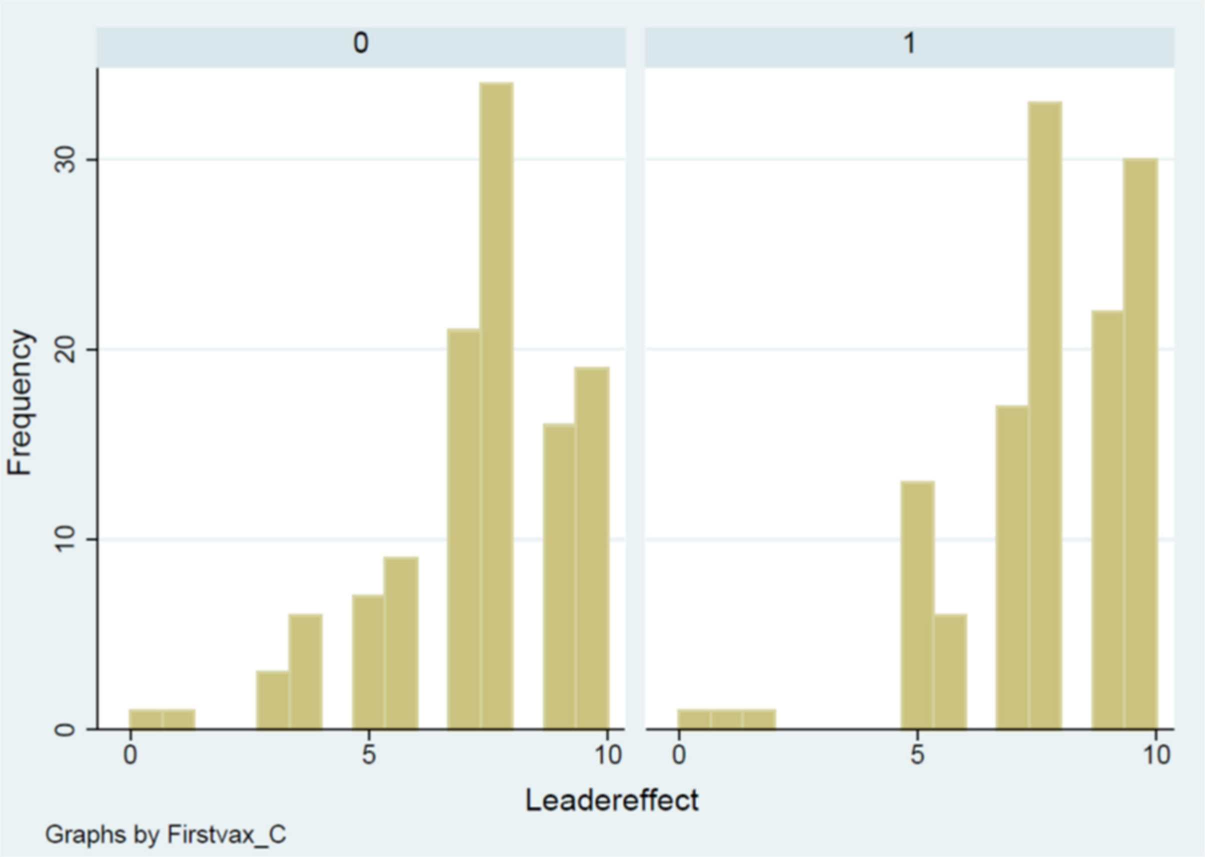

Figure S1. Histogram of participants who did not respond to first call for vaccination (coded 0) and participants who did (coded 1) and their views on how effective leadership was in encouraging uptake of vaccine.

Table S1. Qualitative free text responses to reason for not opting for COVID-19 vaccination during the 1st call for vaccination

Themes	Freq (n, %)*
General side effect	24, 23.7
Lack of information	15, 14.8
Concerns over allergic reaction with vaccine	11, 10.9
Hesitancy with no apparent reason	11, 10.9
Pregnancy, breastfeeding and family planning concerns	7, 6.9
Long term side effect	6, 5.9
Skepticism with undercurrent main themes of novel and rushed vaccine	6, 5.9
Family dissuasion effect	3, 3.0
Efficacy concerns	3, 3.0
Fear of the uncertain	3, 3.0
Concern on interaction with pre-existing disease	2, 2.0
Misc#	7, 6.9

*net number of datapoints N=101; each participant can have inputs coded into 1 or more themes

#vegetarian (n=1), felt pressed for decision (n=1), new hire (n=2), wanted to wait and see if vaccine offer any realistic benefits in reducing routine COVID-19 PCR testing, temperature recording or similar mitigation measures (n=1), perceived lack of support financially and medically in the event of ADR from vaccination (n=1), job relocation (n=1)

Table S2. Qualitative free text responses to concerns on long-term consequences of COVID-19 vaccination

Themes	Freq (n, %)*
Long term side effects in general	25, 19.7
Family planning, pregnancy, BF related issues and effect on offspring	17, 13.4
Fear of the uncertainty in future side effects	16, 12.6
Vaccine efficacy	13, 10.2
No long-term data	8, 6.3
Genetic mutation	6, 4.7
Need for additional doses of the vaccine in the future with undercurrent emphasis on reliance and anticipated costs	6, 4.7
Hypercoagulopathy	4, 3.1
Immune system side effects	4, 3.1
Impact on routine functionality in community and workplace	4, 3.1
Nervous system ADR	3, 2.4
Carcinogenicity	3, 2.4
Chronic disease and/or medication interaction	3, 2.4
New vaccine	3, 2.4
Rapid vaccine development	3, 2.4
Incorrect reasoning[$]	3, 2.4
No vaccine differentiated measures for those who chose vaccination	2, 1.6
Fear gleaned from social media	2, 1.6
Misc[#]	2, 1.6

* net number of datapoints N=127; each participant can have inputs coded into 1 or more themes

[#] Feeling of helplessness while being in a foreign land (n=1); fear of being ostracized (n=1)

[$] Concerned that vaccination would increase vulnerability to infection (n=1); worry over loss of sense of taste with vaccination (n=1); argued that vaccination was not made mandatory because of a high likelihood of long-term side effect (n=1)

Table S3. Summation of number of channels of communication groups who used random social media users and those who did not

	Personal opinions and forums on Social Media use "No" N=207	Personal opinions and forums on Social Media use "Yes" N=34	*p*-value
Additional other channels of comms excl random social media, median (25th, 75th percentiles)	3 (2,5)	5 (3,7)	< 0.001

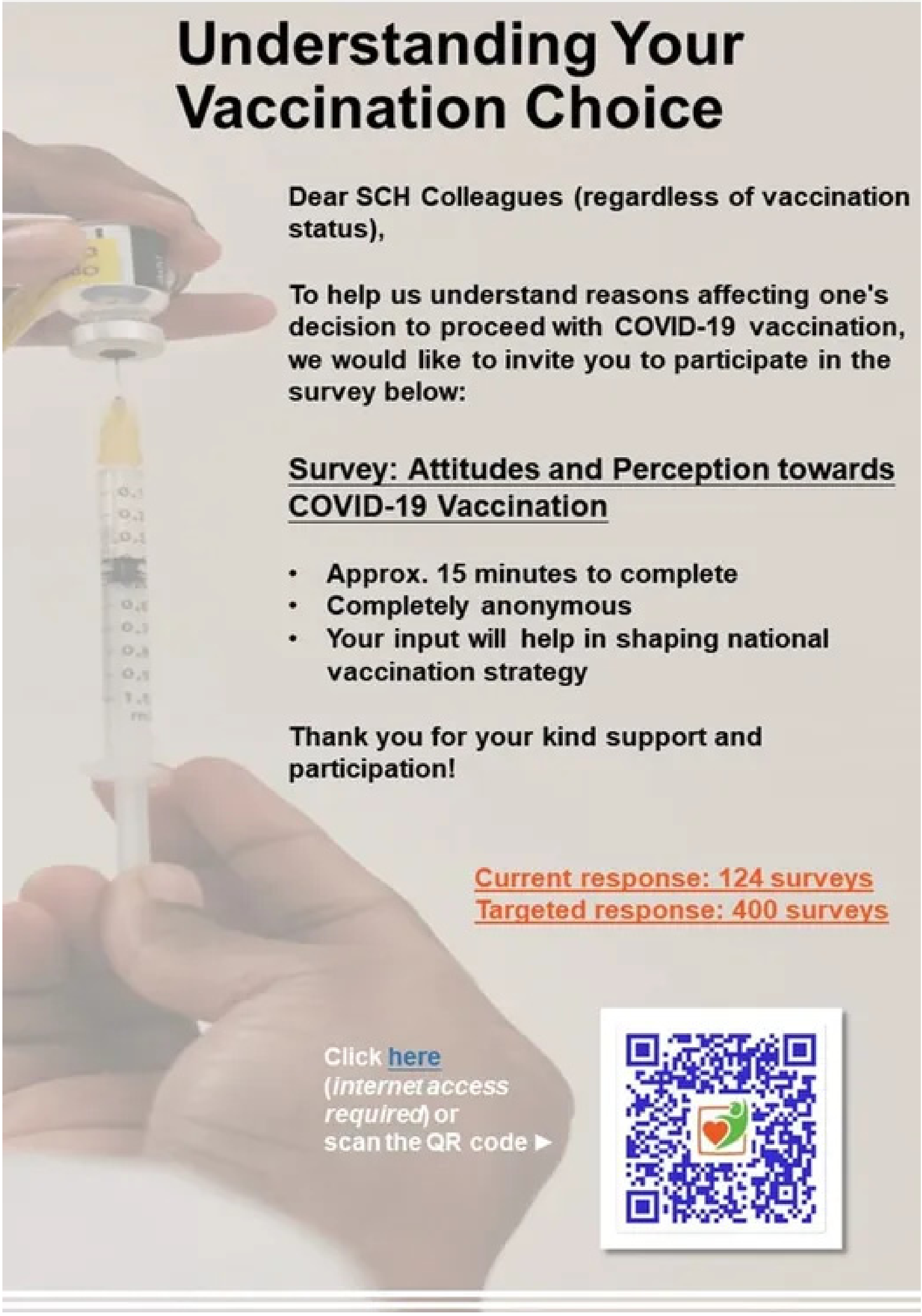

Figure S2. Advertising for enrolment in survey – in the form of posters, email images and hyperlink, online work social platform "Workplace"
